# Exploring the roots of antagony in the safe male circumcision partnership in Botswana

**DOI:** 10.1371/journal.pone.0200803

**Published:** 2018-09-20

**Authors:** Masego Katisi, Marguerite Daniel

**Affiliations:** 1 Department of Welfare and Social Participation, Western Norway University of Applied Sciences, Bergen, Norway; 2 Department of Health Promotion and Development, University of Bergen, Bergen, Norway; TNO, NETHERLANDS

## Abstract

**Background:**

Partnerships in global health and development governance have been firmly established as a tool to achieve effective outcomes. Botswana implements Safe Male Circumcision (SMC) for HIV prevention through a North-South partnership comprising the local Ministry of Health, US Centers for Disease Control and Prevention (funded by PEPFAR) and Africa Comprehensive HIV/AIDS Partnership (funded by the Bill and Melinda Gates Foundation). The SMC partnership experienced significant antagony and the aim of this paper is to illuminate the actions and processes in the SMC program that contributed to that antagony.

**Methods:**

Methods used to gather data include observation of the partners’ planning and strategic meeting in 2012, in-depth interviews with lead officers at national level, focus group discussions with district officers and implementers, younger male officers and old community members as recipients of the service.

**Results:**

The findings reveal that the partnership experienced antagony during operational processes and as the ultimate outcome. Target setting, financial power of the North, superficial ownership given to the South, ignoring local traditional realities results in antagony. Three roots of antagony have been identified: 1. therapeutic domination–medical expertise given with arrogance; 2.iatrogenic violence–good intentions that cause unintended harm; 3. the Trojan horse–Reckless acceptance of the gift as well as deceptive power positioned under the pretext of benevolence.

**Conclusion:**

The three roots of antagony; therapeutic domination, iatrogenic violence and the Trojan horse, constitute attitudes, hidden intentions and unintended consequences that influence program implementation and cause harm at different levels. Examples of therapeutic domination and the Trojan horse have highlighted the need for vigilance at the stage of establishing a partnership, to prevent more powerful partners from developing and applying hidden agendas and to strengthen accountability from the local partner. Iatrogenic violence has highlighted the need for partnership interventions to prevent good partner intentions accidentally producing bad outcomes.

## Introduction

Partnership in global health and development programs has been firmly established as a tool to achieve effective outcomes. It has become a central implementation approach for addressing the world’s complex problems with Sustainable Development Goal (SDG)17 continuing the intentions of Millennium Development Goal (MDG) 8 [1]. The Paris Declaration of 2005 enhances the goal on partnerships by laying down implementation measures, a framework with five principles (ownership, alignment, harmonisation, results and mutual accountability) to ensure aid effectiveness [2]. The partnership model replaces the previously dominant ‘donor-receiver’ model which was heavily criticised for Western hegemony [3]. Partnership is preferred because it is in synchrony with modern values that respect the interests and capabilities of all parties who come together to promote health [4]. Major public health challenges in the Global South–like HIV/AIDS–are tackled by North-South partnerships involving local, national, bilateral government and non-governmental organisations [5–10].

In practice, a fundamental assumption is that partnerships can and should produce synergy: successful outcomes far outstripping what any single organisation could achieve on its own [11]. In addition, contemporary theory predicts that all partnerships–including North-South partnerships for health–produce antagony: some of the burden (the costs) of being a partner may be felt to be excessive [12, 13]. For the purposes of this paper, we define antagony as any resistance within an interaction of two collaborative forces that causes interference, tensions, and counter-productivity. A critical point is that virtually all partnerships should expect to experience both synergy and antagony [14]. Most literature on partnership functioning focuses on investigating determinants of synergistic outcomes,examples [11, 15–19], but little has been done to explore antagony and the underlying processes that shape it.

Partnership theory predicts some antagony and suggests mechanisms for managing a partnership to maximise synergy and minimise antagony. Corbin and Mittelmark [12] used their results for exploring a Global Programme for Health Promotion Effectiveness to develop a systems model they call the Bergen Model of Collaborative Functioning (BMCF). Through this, “the construct ‘antagony’ was introduced as a unique type of output, in addition to synergy and additive results, representing unwanted and disturbing outcomes” Corbin and Mittelmark (12:365). Their findings reveal that antagony can affect partnerships negatively as partners see such investment as a waste of time and resources. Elements that contribute to antagony include power projected by the North over the South, one way communication, unrealistic demands on local partners imposed by Northern partners [12, 13]. However, they also observe that antagony can improve a partnership if partners reflect on disturbing outcomes, learn from their mistakes and work on improving partnership functioning [12]. What is further established is that as much as synergy in health promotion partnerships is both a process and a product [11], antagony is also not only an ultimate outcome but part of partnership processes that keeps feeding back into the collaborative activities. To make North-South partnerships more effective and equitable, research is needed to understand what contributes to antagony as well as: “.. what makes partnership difficult?” [11:418]. This knowledge could be used to improve partnership management. The short-term aim would be to create and maintain a partnership in which all members’ interests and capabilities are valued and respected [11, 16]. The long-term aim would be to help partnerships achieve their public health objectives. Research on both partnership synergy and antagony observes that no matter how successful a partnership is, the partners are almost sure to be dissatisfied about some aspect of the partnership [11, 13, 20]. However in some cases, antagony can be so serious that it leads to premature termination of a partnership [12].

The Voluntary Medical Male Circumcision (VMMC) program in Botswana is locally adapted as Safe Male Circumcision (SMC) [21]. The main partners are U.S. Centers for Disease Control and Prevention (CDC); and Africa Comprehensive HIV/AIDS Partnership (ACHAP) [22–24]. Using qualitative methods, the Botswana SMC program has been studied for lessons to be learned about how to make such partnerships as effective and equitable as possible. In a recent analysis, both synergy and antagony were detected in the SMC program, as predicted by the BMCF theoretical model on partnership functioning [14]. The Katisi et al [14] paper establishes that the model is vague about the underlying processes that shape antagony. The key contribution of the paper to the BMFC theory is that the functioning of the visible in-country partnership is significantly influenced by the less visible global context such as the target setters and donors. The richness of the qualitative data gathered in the SMC study offers the opportunity to explore the underlying causes of such antagony to advance partnership practice and theory. That is the aim of this paper: to illuminate the actions and processes in the SMC program that contributed to antagony and to explore the nature of antagony within the conceptual framework of therapeutic domination, iatrogenic violence, and the Trojan horse.

### Theoretical concepts: Underlying causes of antagony

As to the roots of partnership antagony, three concepts that have been advanced in the partnership literature will be highlighted here.

The first idea is *therapeutic domination* which occurs when the power differentials in the donor-recipient relations are like those in doctor-patient relations, the doctor knowing all and the patient accepting ‘expert’ advice/instruction [25]. Rottenburg [26] describes therapeutic domination as a superior power position taken by those with the biomedical knowhow to manage and direct treatment programs in countries that are poor and vulnerable to such power imposition. He argues that organisations like the President's Emergency Plan For AIDS Relief (PEPFAR) and Global Fund to fight AIDS, Tuberculosis and Malaria (which are seen by the world as holding the superior power of biomedicine) frame disease in terms of absolute emergency, like war and famine, and do not give a chance to address fundamental issues that are crucial to give sustainable solutions. Mawdsley [27] sees aid as a strange gift that is unreciprocated, adopting a lopsided social order where the superior givers do not allow the recipient the dignity of giving something back. Mawdsley [27] calls this ‘negative giving’ that carries ‘symbolic domination.’ The approach that is used to deliver these gifts is individualistic and destroys the local collective action under which recipient communities normally operate [28]. Alden and Schoeman [29] criticise Southern governments for not flexing their muscles strongly like Zimbabwe has done to decisively stand against the imperialistic discourse carried by the North. The same authors observe that it is difficult to balance both meeting local aspirations and expectations of global governance, the latter seem to always take supremacy. “While the language of global HIV/AIDS policy is partnership (ownership, alignment and mutual accountability), the reality is closer to therapeutic domination with donors controlling the nature and extent of interventions” [30:418]. Therapeutic domination shifts the sovereignty power of the indigenous government and gives it to organisations operating at global level, above national answerability [26].

The second idea is that antagony is fomented accidentally; the partners have good intentions, but the complexities of setting up and managing a partnership are demanding, creating unanticipated antagony (Corbin & Mittelmark, 2008). Antagony can also emerge from different value systems and culture, e.g. individualistic approaches cause unintended harm in a collectivist society. Iatrogenesis is a concept from the practice of medicine (meaning injury caused by a doctor) that has been applied to the aid context by McFalls [25] and termed *iatrogenic violence*. This is the term used to refer to unintended antagony in this paper. McFalls [25] uses the term ‘iatrogenic violence’ to demonstrate the political and social disruption that international donors bring into recipient countries while giving benevolent humanitarian services. Usually health intervention programs are declared successful, but long term consequences are only borne by the recipient after the international partners have left, a successful surgery yet a dead patient [25]. Daniel [30] adds that the donor is rather ‘arrogant’ in assuming his solution is the best solution for the recipient, that his solution works, but if it does not, the blame is on the recipient.

The third idea, aid as a *Trojan horse*, conceptualises aid as a deceitful strategy (though accepted willingly by the recipient) to gain control of the recipient country’s approach to health. Antagony arises from the strategic exercise of power, by a more powerful partner, with a hidden agenda to control the partnership. Fowler (31:7) uses the metaphoric phrase ‘Terminological Trojan horse of partnerships’ to describe the subtle power of the international donors from the North who come into the Global South with the deceptive tactic of empowering the weak and seeking to share responsibility of reducing disease burdens but instead bring destruction in many ways. He describes partnership relationships as an aid to foreign penetration, a subtle form of external power imposition [31]. Like with the Trojan horse, recipient countries receive global health programs as contributions to fight disease, while the same trophy is a deceptive way for donors to gain power and control in a battle situation [32]. Welcoming aid from the North into the Global South allows an infusion of foreign values into the domestic approaches to health and development processes [31]. But this act of welcoming also implies a degree of agency [33]. Critics see partnership within the new aid agenda as an instrument for deeper, wider and more effective foreign penetration into the country’s development choices [34, 35]. But it must be noted that the Southern countries allow and accept it. This perspective on partnership has implications for other aspects of the Paris Declaration, for example, ‘ownership’ and community participation is being questioned by researchers [33, 34, 36].

### The case

The partnership for the Botswana SMC program is an extension of a long term partnership between Ministry of Health (MH) and two international organisations that have been in the country for over ten years, assisting in treatment or prevention of infectious diseases of global concern, HIV/AIDS and tuberculosis. CDC and ACHAP are called Development Partners (DPs) because of their long term relationship with the Botswana government [23].

The partners joined in an effort to implement the World Health Organisation’s (WHO) recommendation that countries with high HIV prevalence and low rate of circumcision embark in circumcising HIV negative men, as a partial HIV-prevention measure [21, 37, 38]. MH signed the agreement with WHO in 2010 to implement SMC nationwide and to reach the set target within the context of the already existing support from DPs on HIV/AIDS interventions [21, 22, 24]. International donor organisations, PEPFAR and Bill and Melinda Gates Foundation, gave massive financial and pharmaceutical support through the DPs to help push the target of the adult SMC program: to circumcise 80% of HIV negative men aged 13–49, making 100 000 men per year, in a five year period [22]. Acceptability study for SMC in Botswana was done in 2003 and its implementation in 2009 while the acceptability study for infant male circumcision was carried out later, in 2010 [39, 40]. Adult SMC received massive campaigns in 2011 while infant male circumcision was done as a routine in the health centers and with choice from parents [24]. There were two approaches used in the implementation of SMC. Firstly, the SMC integration approach was locally initiated by MH in 2009, to make male circumcision (MC) one of the long term services within its health system throughout the country. The integration program included dissemination of information, education on SMC and made circumcision services available at all the health centres on a daily basis. Secondly, the Models for Optimized Volume and Efficiency (MOVE) approach for implementation was formulated by WHO and included immediately in PEPFAR best practice [41]. MOVE was introduced two years after integration (2011) to help push the target. The MOVE approach uses biomedical marketing strategies like demand-creating mobilisation campaigns, television and radio advertisements, and celebrities as program ambassadors to persuade men to circumcise [42]. Dedicated clinics do not do any marketing but are simply open to the public for access of SMC services.

## Methods

The paper uses data from a larger qualitative study exploring the SMC partnership in Botswana. Data were collected over a three year period between December 2012 and July 2015 following major developments in the SMC program at particular times.

### Research sites

There were three research sites selected according to the distribution of participants of interest to the study: Gaborone the capital city where all program officers at national level were stationed; Mochudi village which was chosen because it is one of the communities in Botswana that practices traditional initiation which includes circumcision, and also because it is urban and located only 40km from the capital city; and Hukuntsi village which is a community that does not practice traditional initiation and is in a remote rural location.

### Participants and recruitment

The first author observed a three-day SMC annual planning and strategic meeting in 2012 attended by thirty officers from MH and the DPs. The meeting helped inform the direction of the study, selection of participants and motivated the observation of the program over a three-year period. Following the meeting, the first author made direct contact with three officers from MH, one from ACHAP and the one from CDC. The required protocol to inform responsible top managers was followed in order to access willing officers and implementers from MH, DPs and DHMTs for further participation in the research. The following cluster of participants took part at community level: traditional leaders from the two communities; a team of young professional social workers from Hukuntsi village; a team of workers for the MOVE project in Mochudi.

### Data collection

Data were collected using three different methods: Non-participant observation during the 2012 SMC annual meeting; interviews with key officers and traditional leaders; and focus group discussions (FGDs). MH participated in more interviews than the partner organisations; one key officer in MH was the only person to be interviewed three times at different phases of the program because officers from the DPs were unavailable on repeated requests and numerous attempts to follow-up. At community level the only interview was with the chief in Hukuntsi. A total of five FGDs were conducted: Two FGDs comprising five and nine participants in Hukuntsi and Mochudi respectively were conducted for officers managing the program at district level (DHMTs). The third FGD was conducted with a MOVE dedicated team in Mochudi comprising seven officers; the fourth one was with a group of six social workers indirectly involved with SMC in Hukuntsi and the fifth was a group of 25 traditional leaders in Mochudi village. We appreciate that this was too large to be called a focus group, however traditional protocol required that they all participate at the same time even though about ten were most active in responding, giving rich data. The main topics that were explored during data collection included establishing the mission of the partnership; resource contribution; approaches used for implementation; the functioning of the partnership including planning, reporting and communication; the interaction of the program with traditional practices; community response to the program. Data for this paper are largely drawn from the 2012 meeting, interviews with the five national lead officers, the MOVE team and DHMTs as they deal with the partnership directly.

### Data analysis

An audio recorder was used to capture interviews and FGDs but not the partners’ three-day meeting where the first author was permitted only to take detailed written notes. Research assistants helped transcribe the data while the first author cross checked the recordings and translated them into English as necessary. Data were analysed using Thematic Network Analysis, following the steps of Attride Stirling [43]. We used NVivo 10, a computer software package designed for qualitative data management and analysis. Data for this paper were analysed in two phases. The first phase used an inductive approach. Data were coded; related codes were grouped into basic themes which in turn were clustered into organising themes. Finally four global themes were identified, giving four types of antagony. The second phase of analysis was deductive, using the three theoretical concepts–therapeutic domination, iatrogenic violence and the Trojan horse–as the frame to identify roots of antagony.

### Ethics

Ethical permission to conduct the study was obtained from Norwegian Social Sciences Data Services and Ministry of Health (MH) in Botswana. CDC and ACHAP allowed access for research through the MH permit. Informed consent was obtained from all individuals participating. However, the chief in Mochudi gave consent on behalf of the 25 elders, as per traditional protocol. Confidentiality was assured to all participants. However, anonymity of organisations was not promised given the small number of organisations involved.

## Results

Antagony in the Botswana SMC partnership was experienced at two levels: firstly, during the collaborative process and secondly as the ultimate outcome of the partnership. The general findings are presented according to the global themes **in Table 1** showing antagony during the collaborative process. The global themes are: 1. Antagony around approaches to the mission; 2. Antagony around local leadership and commitment. 3. Antagony around financial power and ownership; 4. Antagony around the target. Therapeutic domination, iatrogenic violence and the Trojan horse are viewed as the roots of the antagony that was the ultimate outcome of the partnership. Attempts to analyse data using the three concepts proved to be complex. The three concepts are entangled within the different global themes; therefore they are applied deductively to the findings illustrated at the end of this section. The analyses below are presented first under the four global themes above and at the end of this section we show the entanglement of the three concepts using two examples.

**Table 1:** Themes emerging from data analysis.

Basic Themes	Organising Themes	Global Themes Types of antagony
1. Government health centres blamed for engaging DP-deployed doctors in other medical duties 2. DHMTs believed sharing manpower between MOVE and government staff 3. Integration approach blamed for lower numbers	Tensions around integration approach	Antagony around approaches to the mission
4. MOVE seen as more powerful than integration yet caused dissatisfaction at all levels 5. MOVE undermined equity 6. Lack of resource support from government for MOVE implementation 7. MH reported that MOVE created dependency	Tensions around MOVE approach within the partnership	
8. DPs used different reporting systems than MH’s 9. DPs did not report to MH systematically 10. Reporting between partners was not transparent	Tensions around different reporting systems	
11. Community resisted biomedical marketing approaches used by MOVE 12. MOVE seen as sexualising SMC 13. MOVE advertising accused of being insensitive about male organ 14. Clashes between MOVE approaches and traditional initiation protocols	Tensions around MOVE approach within the community	
15. National leadership viewed as unsupportive of SMC 16. MH’s placement of coordination leadership queried 17. DPs viewed their knowledge of coordination as superior to MH’s	Commitment of local leadership questioned	Antagony around local leadership and commitment
18. DHMT staff commitment queried by DPs 19. DHMTs prioritised attending ill patients above circumcision 20. Health centres viewed SMC as DPs’ program	DHMT’s commitment to program queried	
21. Ownership seemed linked to level of financial contribution 22. MH’s role as owner–with less funds–queried 23. MH’s structures and capital contribution disregarded	Financial power defines ownership	Antagony around financial power and ownership
24. MH viewed as parent when reporting to OECD 25. MH viewed itself as baby not ready to be weaned off support	MH’s role confused	
26. Target (circumcise 100 000 men a year) point of contention as never reached 27. WHO blamed for use of mathematical models against local realities 28. MH blamed for accepting unrealistic target	Target setting questioned	Antagony around the target
29. Donors kept sending more equipment in site of low numbers coming 30. Inconsistencies on balance sheet for used/wasted/ remaining instruments 31. MH blamed for not taking care of surgery equipment	Irony in equipment supplied in relation to public demand for SMC	
32. Financial support tied to target achievement 33. DPs seemed to experience more pressure to meet target than MH 34. All partners agreed to cut target as financial support reduced 35. MH responsible for accounting to WHO for target cutting	Target changed locally	
36. DP2 pulled away employed doctors gradually from 2013 leaving gap in implementation 37. DP1 pulled away financial and technical assistance abruptly in 2014	Target not met–donors pull away	

### Antagony around approaches to the mission

As mentioned above the first SMC approach was the MH’s indigenous approach called integration which was implemented in all health centres in Botswana by government health workers within their normal work routines.

#### Antagony around the integration approach

The integration approach was blamed for slow progress and small numbers at a time.

One DP2 officer said:

Where there is integration there are less numbers achieved

There were tensions around shared staff resources and lack of speed of the integration approach. DPs deployed doctors to different Government health centres to help in implementing the integration of SMC. However, clashes arose about how such staff resource support was used by DHMTs and health centres. One DP2 officer explained:

*When our medical teams were deployed in the health centres to help in SMC integration, they were used on other medical duties. We moved them to focus on SMC as was always intended*.

DHMTs believed sharing manpower between MOVE and government was a form of support One DHMT officer explained:

*But, you know, there are many other things we do in the hospital and we would ask them to help attend sick patients and do other medical duties when they had no clients for circumcision*.

Another DHMT officer added:

Government staff also does SMC. We thought it was a way of supporting one another

#### Antagony around the MOVE approach within the partnership

The MOVE approach was seen as more effective in achieving numbers than integration yet caused dissatisfaction at all levels. Examples follow.

The MOVE approach was seen as undermining equity. One MH top leader showed the need to distribute MOVE resources equally around the country:

*Our talk should be equity, equity, equity in this meeting…to cover all districts with MOVE. Not just a few districts as it is now*.

Tensions around the MOVE approach centred on lack of resource support from government. The MOVE teams were given their own space in government health centres or had separate clinics provided by DPs in some cases. One MOVE team officer said:

*The DHMT support us with structures, transport and equipment… They try, but there are times when we are really busy in our SMC unit and when we seek help from government staff they refuse. They say they have other duties to focus on. It seems SMC is only ours to do. It’s hard to share resources*.

#### Antagony around use of different reporting systems

There were tensions around use of different reporting systems. During the strategic planning period between 2007 and 2009, the partners developed training, reporting, implementation manuals and protocols together, some of which were adapted from WHO. However, at implementation DPs were blamed for following their own separately developed training and reporting systems, causing inconsistencies and undermining MH’s coordination. One of the MH lead officers at the three day meeting said:

*We do not have one comprehensive reporting system. Partners report their own way, the ministry reports in its own way. Even the DPs’ ways of reporting are not the same*.

The MOVE teams were supposed to report to the DHMTs, who in turn would report to MH. The 2012 Monitoring and Evaluation report at the three-day-meeting showed that there was only one DHMT receiving such reports from its MOVE team. One MH officer said:

*We wonder where the MOVE teams were reporting, if not to MH*.

The officer presenting explained that some districts’ reports were only captured via DP1 or DP2 brief reports at the national technical committee meetings. One DP1 officer responded to this:

*You see demand creation is housed outside the Ministry organisational level. It is not the Ministry doing demand creation. It is partner organisations*. *How then will the DHMTs feel ownership of the report even if the teams would report to them?*

An MH officer suggested:

*There is a disconnection between MOVE teams and DHMT*. *We should address this*

There were disagreements on the issue. One DP2 officer added:

*If universally there is zero DHMT reporting, it means the leadership has failed. I disagree that there is a proper system within MH. The DHMTs do not know what is expected of them with regard to demand creation and to reporting. So it is an incomplete system*.

The assistance from MOVE teams was seen as both a great help but also said to create dependency. An example of what most MH officers said follows:

*..while we benefit a lot from partners through MOVE…, at the end of the day they are creating a dependency syndrome. Our staff doesn’t have as much time to work for MOVE only…. districts are overwhelmed with many other programs*.

#### Antagony around the MOVE approach within the community

The communities did not appreciate MOVE’s biomedical marketing approach. A team of social workers asked about their *personal* views regarding SMC expressed that the public advertisements sexualise SMC. The following quotation represents what many said:

…It is all about sex…they say you will look cool when you are circumcised…you will enjoy sex more than before…MC is just sexualised

Although the SMC celebrity (contracted as the ambassador for the program) was applauded for being a crowd puller, his publicity talks on SMC were not liked. One social worker said:

*…You see he* [the celebrity] *only emphasises the sexual enjoyment part…being attractive to women*. *That is why young men circumcise*. *They want to be attractive to women and be promiscuous*.

MOVE advertisements were accused of being insensitive in several ways. Firstly about the male private organ: Traditional leaders suggested that MOVE should not use words like the ‘penis’ when formulating advertisements. Second, there were clashes between the MOVE approaches and traditional initiation protocols. The quotation below, from an elderly man in Mochudi, represents the traditional voice:

*..You see they do not want to come for SMC. Do not force them. They want to answer to the call for initiation by the chief first*.

Besides the cultures under study, other communities practicing initiation had a challenge with SMC conflicting with their traditional protocols. The Baherero culture of Boteti communities silently rejected SMC by not participating in it, instead, quietly carrying out their ways of circumcising. The Barolong chief of Goodhope village intensely recruited men for initiation, to oppose SMC requirements that were seen as mystifying the culture. The Ramotswa traditional leadership of the Balete culture abhorred SMC advertisements that MH and DPs made against their will, when they had invited them to circumcise their initiates in the wilderness. There were also complaints about women medical officers conducting the circumcision as well as use of public space like clinics when circumcision was regarded as a secret male rite. Although SMC officers expressed wishes to reconcile mobilisation approaches with community expectations.The MOVE approaches to advertise the program took supremacy.

### Antagony around local leadership and commitment

The DPs questioned Government leadership’s commitment and priorities at both national and district level. The national leadership was seen as aloof from the program: not owning it and advocating for it like it did other HIV intervention programs such as Prevention of Mother To Child Transmission (PMTCT). A DP2 officer said:

*The leadership should give a public face and support to the SMC program but they are not* .. *they did with PMTCT*

Meanwhile the national traditional leadership was also not impressed by the misuse of initiation phrases, making public demonstrations against MH for this.

MH national leadership was also questioned for not physically placing its regional coordinators closer to their on-ground operational regions (North and South). MH expressed a wish to be trusted on its decisions about coordination of the program. One MH officer reacted to this:

This is not the first time I hear this …will you be happy if I tell you how to structure and run your organisation?

One DP1 officer explained that their organisation brought in skills to teach government on how to operate partnerships and on higher roles like coordination of the program.

DHMT staff commitment was also queried by DPs. They reported that it was their MOVE teams only that were committed to SMC. The quotation below represents how many DHMT staff responded to this:

*…a sick patient is more crucial to attend to than clients for SMC…We appreciate that the DPs’ MOVE teams can focus on SMC because we have many other duties to do.…but we support them with resources and we help where we can*.

Antagony around financial power and ownership

#### Financial power defines ownership

All MH and DP officers acknowledged MH as the owner of the program and a manager for all health activities. However, financial power seemed to define ownership of the program. Partner roles were clear; with MH seen as the owner of the program and DPs as supporting partners. However, there was contention around evidence of ownership. Ownership seemed to be measured by the level of financial contribution. One comment by a DP officer seemed to imply that their level of resource contribution actually made them the owner of the program. The officer said:

*So if I’m putting more resources than MH on the table but I get not to own the program what am I*? *They own the program but they are not putting their own resources …I think it has been a very tense relationship between us in the past but I think things have gotten smoother*.

The same officer said:

*I really wish the government owned the program truly as their program, not outsource it as the implementation reveals now… The Government of Botswana has not budgeted to achieve this without donor funding*.

One MH officer explained the government’s stand when this was debated at the three-day meeting: He responded to the query this way:

*Government budget is about 5 million pula (666 667 USD). This is specifically programmatic funds. Apparently DPs’ funds also cover overhead costs etc, but government has all its overhead costs covered already because it uses its employees, equipment and existing structures. Comparing figures then would confuse*.

#### MH’s role is confused

Although the DPs were blamed for not reporting transparently and consistently to MH, the Government of Botswana was responsible for reporting on general financial and implementation performance (representing all partners) to OECD [Organization for Economic Cooperation and Development] regardless of limited information. One MH officer explained

DPs do not tell us everything about their budgets…The Minister has to act like a parent and cover this even though we are not informed about everything concerning DPs budgets distribution…how much they use for capital expenditure

In contrast to taking responsibility like a parent, at times, MH regarded itself as still dependent. For example when donors were threatening to decrease funds one officer used the expression

..you cannot wean the baby..

Antagony around the target

#### Target setting is questioned

The mission of circumcising 80% of HIV negative men in five years, which translated into a target of 100 000 circumcisions a year, became a point of contention as this target was never met in all the years of the program’s existence. There were debates on who set the target and why government agreed to it, yet all entered the partnership aware of the mission and the target. A technical officer from MH explained who set the target:

*WHO used mathematical models to help Botswana set the target. The questions asked by WHO for the setting of the target were like: how much of the coverage do you need to have impact on protection..? Calculations were made based on the expected impact*.

The house at the 2012 meeting agreed in unison that the target set for Botswana was unrealistic. The quote below represents voices of all partners:

*..but the target looks crazy, ..It’s ambitious*.

The target was brought forward as a major risk of program failure by all 30 participants at the three-day meeting. The house blamed WHO for just using mathematical calculation and not relating that with realities on the ground. They also questioned why Government accepted such high ‘unachievable’ target. One DP2 officer said:

Is Botswana being realistic? From the word go, we have already been sent to fail before we start. We are now at 14.7% of the target we hope to achieve by 2016. What are we saying?

Another DP1 officer added:

*Botswana government accepted the target. And it’s a self-fulfilling prophecy in my opinion because you agreed to the target which you are never going to attain*.

#### Irony in SMC public demand and equipment supply

There is inconsistency between public demand for SMC and the supply of equipment. Although the target was hard to reach, DP1 still brought in circumcision surgical kits from pharmaceutical companies abroad. It was reported that close to 100 000 kits were received but less than 40 000 were used. Government health centers were blamed for underutilization and misuse of the kits as the balance sheet reflected.

It seemed pressure to meet target was experienced more by the DPs while MH preferred long term program development within its health system. One MH officer explained:

*You see with government, it is about ensuring that the service is permanently established within the government system. The DPs are experiencing pressure to meet donor expectations. So.. we understand their frustrations. MH also strives to meet the target but the most important thing is long term establishment of the program*.

#### Target is changed locally—Working against WHO

During the 2014 round of interviews, several officers reported that MH and partners had taken a decision to cut down the target to circumcising 50 000 instead of 100 000 men per year. However they also expressed fear that by lowering the target it conflicted with the WHO recommended target and expected impact on infection rate. One MH officer explained this dilemma:

*We have cut down the target to 50 000. It is realistic to us given the circumstances, but it will not help us meet the 80% infection reduction rate… We will have to explain this to WHO. Their 100 000 target still has not changed*.

At the time the target was revisited in 2014, the DPs had already started reducing their financial contribution to the program. One DP1 officer explained:

*DP2 is now chipping in 25% of the 100,000 target per year at the moment instead of 40% as it was initially. We were also hoping to chip in 25% or more but we have to cut it to 15% just basing on reality. Our donors are not happy about the low numbers*. *They have cut down funds…*

#### Target not met—Donors pull away

Donors ceased their participation in the program between mid-2014 and 2015. After the donors pulled away only one officer was able to give us access for further interview. Whereas MH explained the pulling away of the DPs with a sense of empathy there was at the same time, an expression of despair within the MH. The MH officer said:

*They have pulled out at a very wrong time. In fact, it is a loss on our side. It was unfortunate because there was no consultation when they were deciding to pull out. They just notified us in less than a month to say all the support from DP1 to Botswana SMC will be terminated. So, most of the contracted partners that we had that were funded by DP1 are no more*.

The officer explained that he pulling away of DP1 hit MH hard.

*Last year we lost DP1 that was supporting the bulk of the program**…**because they were becoming agitated that they were spending a lot of money but the results were not coming, so they pulled out. DP1 is America itself… …Obviously this has already shaken us. When they pull out they withdrew a promise of billions of US Dollars. Where will we get that money? DP1 had contracted many other partners. So they are gone*.

The MH officer explained that they were experiencing the gap left by DPs’ medical teams and that the government could not manage the gap at that time, therefore men seeking SMC service at the dedicated clinics were leaving without being helped. After the partners pulled away MH planned to continue the integration program. They planned to revisit their implementation strategy and involve the community more so that the community approaches could be used to recruit men.

## Conceptual analysis

Attempts to analyse data using the three chosen theoretical concepts; therapeutic domination, iatrogenic violence and the Trojan horse proved to be complex. Examples of each concept can hardly be represented by a single quotation but is seen throughout the four global themes in the findings. The antagonistic outcome is also rooted in the three concepts. Below we demonstrate the entanglement of the three using two themes, MOVE and the community. Our two themes represent two levels of interaction: Interaction between MH and the DPs and interaction between the partnership and the communities.

### The MOVE project as therapeutic domination, iatrogenic violence and the Trojan horse

The MOVE, of itself, is an act of *therapeutic domination* by the North. Designed and created by WHO and adopted by PEPFAR, it carried with it the arrogance of knowing best. The approach of the Global North dominated over the local integration approach, over the culture and traditional views on how things should work. However, the government of Botswana allowed the DPs to take the initiative and lead the MOVE activities. Botswana’s relaxed attitude and DPs domination created community resistance. Regardless of the resistance, and without seeking to learn from antagonistic tensions with communities, external approaches continued to dominate. When expectations were not met, partners pulled away. The North disregarded the responses from the local communities and did not even revisit its approach. Although the need to listen to local communities’ views was discussed in meetings as important, it was never made a priority. The MOVE has in it *iatrogenic violence*. We observe that it was brought in intended to do good. But when things went wrong–like the purchase (by partners) and waste of equipment–MH had to take responsibility. Likewise when the partners pulled out MH was left with the responsibility of equipping and managing the dedicated SMC clinics that had been set up. This is an act of a doctor starting medication and then withdrawing it—bringing harm instead of healing. The MOVE project was also a *Trojan horse*, a false ‘gift’ from the North presented as a solution to push the set target to circumcise 100 000 HIV-negative men in a year in Botswana. MH was happy to receive it since its ideas and strategies appeared good on the surface. However a closer look reveals that MOVE ‘carried hidden destructive motives’: of acting powerful and side-lining the indigenous approach–integration; operating as a separate entity and creating dependency instead of building on the Botswana health system that exist to improve it; pulling away and leaving MH with no resources. The irony here is that MH accepted the “gift” and even contributed to the partnership. The DPs are more tied to donor driven expectations regarding outputs than the ministry of health.Therefore MH could be held accountable for not standing strong to ensure that the voice of the community is heard and that integration of services as well as the desired indigenous approaches are followed.

### Interaction with the community as therapeutic domination, iatrogenic violence and the Trojan horse

The SMC program promoted medical circumcision as ‘better’ than traditional circumcision within initiation. Communities were expected to integrate SMC into their traditional practices but the views of communities were neither listened to nor their values respected–especially regarding circumcision within initiation. The community experienced *therapeutic domination* from the SMC program. In fact, even beyond a lack of respect for their values, their social and cultural customs were disturbed, for example through the use of women conducting the circumcision operations. This disruption represents *iatrogenic violence* which was exacerbated by the introduction of MOVE. In response to resistance by the chiefs, MOVE sought ways to recruit men and boys without the chief’s support, for example by campaigning in schools or with a popstar ‘ambassador’, thus finding a way in behind the community defences–just like the *Trojan horse*.

## Discussion

This paper has explored different roots of the antagony experienced within the SMC partnership in Botswana. Antagony around finances, leadership and ownership, the local and international approach to implementation, community’s responses regarding the MOVE’s biomedical marketing approaches, are all a reflection of the behaviour of the North on health partnerships. We use Fowler’s [31] and McFalls’ [25] descriptions of such behaviours to expose the underlying processes of such tensions.

### Therapeutic domination

Partnership implies an equal, respectful collaboration in achieving a common goal, but the inequalities in roles and status of the partners caused tension and confusion. The MOVE approach carried with it Northern domination and control, proving to be powerful while diminishing local approaches and the local partner. The MH seemed to have uncertainty concerning its position as the owner of the program, and its ownership was also questioned and doubted by the DPs. The MH presented itself with three metaphorical descriptions at different times: as the owner, the parent and the baby. In view of itself as the host, beneficiary and health systems manager MH claimed the position of the “the owner.” It also called itself “the parent” or “parental state” that signed the agreement with WHO and has the obligationto report country performance and financial stand to OECD. The irony is that it did not know all the financial undertakings of the DPs since transparency was limited. When the DPs were threatening to pull out it claimed the position of a “baby that should not be weaned.” In contrast, the development partners gave Botswana the title of “owner” yet labelled it as slow to push its own target; they accused MH of professing ownership yet not acting as owner. Also, the highest leadership of the country was criticised for not supporting the program as its “face.” Therapeutic domination by donors gives ambiguous roles to the local partner, calling them “owners” of the program, but treating them as less-able recipients who should follow rather than lead [25, 44, 45]. This domination leaves the recipient countries unsure of their status in the partnership [30]. Critics argue that the fancy leadership/ownership phrases given the recipient countries are just a facade to cover the continuous exercise of power by the international financial agencies [46]. Indeed financial power makes survival of aid partnerships grossly depend on the decisions and interests of the international donors [46]. This is because international agencies see themselves as experts. Recipient countries in turn endorse external new and exciting programs that they may not have the capacity to sustain [47]. This is because they see the donor as ‘expert’ as a patient might view a doctor. The implication here is that while the language of global HIV/AIDS policy is partnership, the reality is closer to therapeutic domination with donors controlling the nature and extent of interventions for the “good” of the recipient [25].

In practice ownership was demonstrated through financial power. Financial resources, implementation speed and the social marketing approach made the international partners seemingly more efficient as they effectively took control of the implementation, mystifying the ownership of the program. However government health workers were also overwhelmed with other duties and had little space for SMC. The development partners insisted to know MH’s financial contribution, hardly giving acknowledgment to MH’s contribution of infrastructure and human capital. Instead, they viewed themselves as doing better than MH on financial contribution, yet without being called “the owners”.

There is another contra observation in this partnership: Although in practice, ownership was demonstrated through financial power, the balance of power here seems more delicate than financial contribution. MH had given in-kind contribution through infrastructure, human resources, supply chains, policy and overheads. Therefore, instead of being powerless against the DPs and donors, the MH shifted the burden of SMC scale-up (and particularly MOVE) both in terms of finances and other resources on to DPs. Alden and Schoeman [29] argue that accepting the “gift” is a sign of agency and comes with responsibility. Therefore blaming the DPs does not take away the responsibility of the local partner. As a “parent” MH could have given more voice to their citizens and traditional leadership.

### Iatrogenic violence

Partnership intends to do good and to produce harmonious results but in this case it also caused unintended harm. Iatrogenic violence was experienced at different levels [25]. The severity of the HIV epidemic generates a need for external help. MH appreciated the support from WHO and the development partners, seeing that the country alone would not manage the target oriented program [24]. Indeed the MOVE approach helped achieve greater numbers even though the set target was not reached. External partners usually bring gain to recipient countries: skills, human and capital resources, and expertise on operations [33]. On the contrary, as McFalls [25] argues, humanitarian intervention on a genuine existing problem can cause havoc instead of help, the harm being unintended. There are several examples of such unintended harm in this case. Firstly, MH observed that external help created dependency at national and district level. The DPs complained that the DHMTs were not prioritising SMC and were only dependent on the MOVE teams to do all the work. The creation of dependency and stripping local people of their agency is a form of iatrogenic violence [30]. Secondly, the abrupt pulling away of donors’ resource and implementation support caused operational disruption, “the pain” and the “violence.” One officer explained that whereas DP2 had been downsizing support slowly, with minimal effects, DP1’s pulling away its massive resources was sudden, within a month’s notice. This caused a sudden collapse of the activities of the program because DP1 was the main source of support to both local and national level. It was the donors behind the DPs–PEPFAR and Bill and Melinda Gates Foundation–rather than the in-country DPs, who were seen as the perpetrators of this iatrogenic violence. The MH officer identified international donor organisations’ sudden cutting off their financial power as the root of the problem and expressed that the DPs had no choice but to pull out. McFalls’[25] conclusion that the governing of humanitarian aid is a dictatorship (all-be-it a benevolent one) that is unfortunately exempt from the challenges and critiques of normal politics. He contends that no matter what the nature or context of the intervention ‘iatrogenic violence is inherent in the formal structure of international intervention’ [25:320].

### The Trojan horse

Partnership implies transparent collaboration to achieve a common mission but in SMC the agenda of the Northern partners seems hidden in the term ‘partnerships.’ The North is accused of having a hidden agenda to control, of practicing foreign penetration and imposing power [48]. Fowler [31] calls this ‘the terminological Trojan Horse of partnership.’ Several authors add to Fowler’s description in different ways. Guilhot [49] describes external agencies as pretending to be ‘catalysts’ under the pretext that they are quickening processes to achieve locally set goals, yet exerting their external control. Abrahamsen [35] argues that external partners appear kind and claim to be on the back seat, yet they use indirect forms of external control to direct the recipient countries’ health development choice; pushing their own agenda rather than that of the local Government. St-St-Pierre [48] cites an example of how humanitarian aid brings harm to recipient countries instead of helping, that: when countries like Nigeria decreed a lawsuit to freeze clinical trials, Pfizer biopharmaceutical company alluded that no help will be given to Nigeria if the country encounters another epidemic outbreak.

In this partnership, MH’s approach of SMC integration into the national health systemwas “overpowered” by donors’ technocratic accountability mode of dollar-to-numbers that brought in the “MOVE project to push numbers towards set targets” as one DP2 officer explained. The MOVE project brought in American values–the approach of biomedical marketing that targets individuals, as opposed to the collective approach preferred by the communities receiving the service [42]. Foreign penetration is evident on the US pharmaceutical companies pushing their commerce agenda [48]—supplying surgery kits in large quantities regardless of the low response from men. It turned out that MH was scapegoated for lack of use of surgery kits. WHO and the World Bank recommend that countries receiving aid be given the liberty to make choices and work out their own objectives [1, 33, 35]. The same WHO that recommended consideration of culture and indigenous ideas during implementation of programs, demands reports that focus on numbers while and not capturing local responses [42]. These findings characterise how the domineering power of the North over the South is concealed [31]. Abrahamsen [35] contends that it is a struggle to attain genuine partnership based on fairness and mutual respect under circumstances where one party owns and controls the purse and the other the begging bowl.

The MH is not blameless. The Southern partners’ are responsible to ensure that their voice is heard and not silenced by their lack of financial power and expertise [29]. The MH’s voice;—to advocate for equity; to pursue and meet traditional expectations; to show off its financial contribution in the form of infrastructure; to address their query on incoherent reporting; to refuse unequal accountability; to ensure a realistic view on target against WHO target—was not strong enough and therefore easily suppressed by the DPs’ zeal to achieve target. MH accepted the target from WHO when agreeing to carryout SMC [21, 24]. Both MH and DPs felt pressured by the donors and WHO to fulfil the target hence they compromised genuine consultation with communities (which takes time). But it was in the MH’s power to lead the implementation in a way that represented community views. It seems negotiations with WHO are possible [42]. Lesotho is one example of a country that prioritised its traditional views regarding SMC instead of rushing to sign an agreement with WHO when not prepared to reach target. Nonetheless, Lesotho was seen by UNAIDS as non-compliant, against universality and “recalcitrant” [50:766]. What is known is the new global health agenda allows renegotiations and compromise [50]

The nature of antagony in the SMC program in Botswana has implications for North-South health partnerships theory and practice. As to theory development, the findings of this study suggest roots of antagony that partnership planners and managers should be alert to. As to practice, evidence for iatrogenic violence has highlighted the need for partnership interventions to prevent partners’ good intentions accidentally producing bad outcomes. The metaphors of the Trojan horse and therapeutic domination have highlighted the need for vigilance and rigour at the stage of establishing a partnership, for the local partners to take responsibility and prevent more powerful partners from developing and taking over local implementation. As an appeal, researchers should consider exploring measurement of antagony in partnerships just as they have done for synergy.

## Limitations

It is acknowledged that the purposive selection of participants may not represent all views in the larger community of Botswana. Although the research covered a variety of participants, most data used in this paper come from the national and district officers and other data were used to validate their statements and observations. Voices of young men from the communities were not represented in this paper. The other limitation is that the voice of donors is not heard since donors were not interviewed. Although this paper may be used as a learning resource, the exploratory nature of this study makes it impossible to generalise the findings.

It is unclear from this analysis what might distinguish countries that experience less antagony and make the partnership work better, and those countries that experience antagony that affects the partnership more severely. An independent in-depth on this will contribute to showing what it means for partnerships going forward, not only in Botswana but in the rest of the region.

## Conclusion

This paper has explored different types r of antagony and their roots. Target setting, the financial power of the North, superficial ownership given the South, ignoring local traditional realities resulted in antagony. In spite of the rhetoric of partnerships, the continued domination of the northern partners has been illustrated by three concepts that influence the outcome of the partnership. *Therapeutic domination*–expertise given with arrogance–was shown in prioritising external approaches such as the MOVE project which was more concerned about numbers than other implementation realities, side-lining the local Government’s approach of integration. The DP’s lack of acknowledgment for MH’s non-financial contribution indicated their belief in the superiority of their modes of involvement. *Iatrogenic violence*–good intentions that cause unintended harm–was evident when MH was left with the sole financial and operational responsibility for all aspects of the program once the DPs pulled out. The *Trojan horse*–deceptive power positioned under the pretext of benevolence–is illustrated by the MOVE approach that brought false hope of victory but was a vehicle for donor control. Receiving the gift and then blaming the giver is a sign of weakness of the South. These underlying attitudes, approaches and intentions all contributed antagony to the SMC partnership in Botswana.

## Supporting information

S1 File (PDF)

## Abbreviations

ACHAP: Africa Comprehensive HIV/AIDS Partnership
CDC: Centers for Disease Control and Prevention
DHMT: District Health Management Team
DP: Development Partner
MH: Ministry of Health
MOVE: Models for Optimizing Volume and Efficiency
OECD: Organization for Economic Cooperation and Development
PEPFAR: President's Emergency Plan for AIDS Relief
SMC: Safe Male Circumcision
VMMC: Voluntary Medical Male Circumcision
