# Supplementary material for: Exploring the roots of antagony in the safe male circumcision partnership in Botswana
Source: PLoS One. 2018 Sep 20;13(9):e0200803. doi: 10.1371/journal.pone.0200803 (PMC6147398; doi:10.1371/journal.pone.0200803)
Supplement: S1 File — (PDF) [file pone.0200803.s001.pdf]

SI File PDF
Supporting Information

Interview guide - national SMC officers at Ministry of Health, CDC and ACHAP

Partnership and Safe Male Circumcision, SMC, General

1. Could you describe in detail what the Safe Male Circumcision (SMC) for HIV prevention is about?
2. Who are the partners in the SMC for HIV prevention?
3. Who among the partners leads the SMC strategy?
4. Could you describe the role of each partner in the program? What is your role ?
5. What kinds of resources does each partner contribute?
6. How are the targets for SMC set?
7. What kind of program activities is each partner engaged in?
8. How does the level of participation vary between the partners in the program of SMC for HIV Prevention?
9. What would you describe as the greatest achievements for SMC for HIV prevention?
10. What would you describe as the greatest obstacles for the success of SMC Program?

Questions on Implementation

11. Can you tell me how the partnership roles impact its functioning?
12. How do leaders at the national\community level support the implementation?
13. How do you measure success of the SMC program?
14. What age responds the most to SMC? Why do you think they do as compared to other ages?
15. Is SMC meeting its targets? In your own view, what makes it succeed? What makes not succeed?
16. What are the challenges to meeting targets?

Focus group guide – DHMTs, MOVE team and Social Workers

1. Can you tell me how the partnership roles impact its functioning?

How do leaders at the national\community level support the implementation?

2. How do you measure success of the SMC program?
3. What age responds the most to SMC? Why do you think they do as compared to other ages?
4. Is SMC meeting its targets? In your own view, what makes it succeed? What makes not

succeed?

5. What are the challenges to meeting targets?

6. What is the view of the community leadership to SMC program?

7. How is the community responding to your different campaign strategies for SMC?

8. Are there any particular aspects within this community\Botswana that facilitate the success of SMC program?

9. Are there any particular aspects within this community\Botswana that inhibit the success of SMC program?

10. According to your observation, what moves\encourages men to be circumcised through SMC?

11. According to your observation, what discourages men to be circumcised through SMC?

12. How is the support of media to SMC?

Round 2 interview guide: SMC national officers at Ministry of Health, CDC and ACHAP

1.Could you update me on the current status of the partnership?

2.What have been the successes?

3.What have been the challenges?

4. What is your view on Peacecorp Volunteer work?

a) What makes it easy for you to work with the community?

b) What challenges do you experience in working with the community?

c) What cultural challenges\clutches has the partnership addressed?

5. Are there any particular aspects within this community that facilitate the success of SMC program?

6. Are there any particular aspects within this community that inhibit the success of SMC program?

7. What difference has the partnership between the three organisations brought to the program that

would have not been(or not been easy) easy to achieve without the partnership

8. What would you describe as the greatest obstacles for the success of SMC Program?

9. What would you describe as your greatest achievement while serving in the SMC Program?

10. What age responds the most to your services? Why do you think they do as compared to other ages?

11. According to your observation, what moves\encourages men to be circumcised through SMC?

12. According to your observation, what discourages men to be circumcised through SMC?

13. How do you measure the success of the SMC program?

14. Do you have any suggestions on increasing the uptake of SMC?

Focus group discussion guide – traditional leaders

1. Are there any cultural practices surrounding male circumcision in Kgatleng?
2. What does Male Circumcision,MC, mean in your tradition, and what does SMC mean?
3. Are there any differences between SMC and traditional initiation?
4. Are there any similarities between SMC and traditional initiation?
5. How do you work with the SMC program officers?
6. How is your community responding to SMC?
7. Are there any aspects of the SMC program that you appreciate?
8. Are there any aspects of SMC that you do not appreciate? Please explain
